# Anti-HIV Activity of Snake Venom Phospholipase A2s: Updates for New Enzymes and Different Virus Strains

**DOI:** 10.3390/ijms23031610

**Published:** 2022-01-30

**Authors:** Andrei Siniavin, Svetlana Grinkina, Alexey Osipov, Vladislav Starkov, Victor Tsetlin, Yuri Utkin

**Affiliations:** 1 Shemyakin-Ovchinnikov Institute of Bioorganic Chemistry, Russian Academy of Sciences, 117997 Moscow, Russia; andreysi93@ya.ru (A.S.); osipov-av@ya.ru (A.O.); vladislavstarkov@mail.ru (V.S.); victortsetlin3f@gmail.com (V.T.); 2 N.F. Gamaleya National Research Center for Epidemiology and Microbiology, Ivanovsky Institute of Virology, Ministry of Health of the Russian Federation, 123098 Moscow, Russia; grinkina.s@mail.ru

**Keywords:** antiviral activity, human immunodeficiency virus, phospholipase A2, pseudovirus, retrovirus, snake venom, syncytium, recombinant form

## Abstract

Since the beginning of the HIV epidemic, lasting more than 30 years, the main goal of scientists was to develop effective methods for the prevention and treatment of HIV infection. Modern medicines have reduced the death rate from AIDS by 80%. However, they still have side effects and are very expensive, dictating the need to search for new drugs. Earlier, it was shown that phospholipases A2 (PLA2s) from bee and snake venoms block HIV replication, the effect being independent on catalytic PLA2 activity. However, the antiviral activity of human PLA2s against Lentiviruses depended on catalytic function and was mediated through the destruction of the viral membrane. To clarify the role of phospholipolytic activity in antiviral effects, we analyzed the anti-HIV activity of several snake PLA2s and found that the mechanisms of their antiviral activity were similar to that of mammalian PLA2. Our results indicate that snake PLA2s are capable of inhibiting syncytium formation between chronically HIV-infected cells and healthy CD4-positive cells and block HIV binding to cells. However, only dimeric PLA2s had pronounced virucidal and anti-HIV activity, which depended on their catalytic activity. The ability of snake PLA2s to inactivate the virus may provide an additional barrier to HIV infection. Thus, snake PLA2s might be considered as candidates for lead molecules in anti-HIV drug development.

## 1. Introduction

Human immunodeficiency viruses (HIV) are two species of retroviruses from the *Lentivirus* genus that cause a slowly progressive disease: HIV infection, at which the work of the immune system is suppressed, and acquired immune deficiency syndrome (AIDS) develops. There are two HIV types: HIV-1 and HIV-2, differing in their origin [1]. During AIDS, a progressive failure of the immune system results in life-threatening opportunistic infections and cancers that are not characteristic of people with normal immune status. Healthy people can get HIV from infected people through the exchange of body fluids (blood, breast milk, semen, and vaginal secretions); the transmission of HIV through the genital mucosa is an important route of HIV infections in humans [2].

At the molecular level, HIV infection occurs through the interaction of the viral glycoprotein gp120 with the cellular CD4 receptor [3] and chemokine coreceptors, usually CCR5 in the early phase of infection and CXCR4 in the later stages of the disease [4,5]. However, HIV can infect cells both as a cell-free virus and also through virus-infected cells that express virus-specific antigens on their membranes and produce viral particles [6,7]. The binding of HIV to its target cells and fusion of HIV-infected and uninfected T-lymphocytes require multistep specific interactions of the viral envelope glycoproteins gp120/gp41 with the cellular CD4 receptor and HIV coreceptor CCR5 and/or CXCR4 [8]. After binding to cellular receptors, the gp120/gp41 complex undergoes conformational changes that promote fusion of the viral membrane with the target cell membrane [9,10]. After virus–cell fusion, virion uncoating occurs [11] to release the reverse transcription complex (RT), which dissociates from the plasma membrane and moves to the cell nucleus [12]. Then, the viral DNA enters the cell nucleus, where viral integrase facilitates the integration of linear forms of pro-viral DNA into the chromosomes of the host cell [13]. 

An effective HIV prevention should be aimed at suppressing the spread of both cell-bound and free viruses. Therefore, new compounds capable of inactivating the extracellular virus and blocking HIV-mediated cell fusion may be promising drugs for protecting cells from HIV infection.

Natural products contain a huge range of compounds with a wide variety of chemical structures and biological activities, which makes them an important source of substances for the development of new medicines. Among such natural products, venoms are complex mixtures, including various components of enzymatic (metalloproteinases, serine proteinases, phospholipases A2, L-amino acid oxidases, etc.) and nonenzymatic (neurotoxins, cytotoxins, disintegrins, etc.) nature, which can provide clues for the development of therapeutically useful molecules [14]. 

Secreted phospholipases A2 (PLA2s) have been found in mammalian tissues and animal venoms and catalyze the hydrolysis of glycerophospholipids with the release of free fatty acids and lysophospholipids [15,16,17]. They have been classified into different types based on amino acid sequences and the number and position of cysteine residues [18,19]. Secretory PLA2s (sPLA2s) comprise more than one-third of the members of the PLA2 superfamily and are subdivided into 10 groups and 18 subgroups. The snake venom PLA2s belong to groups IA (Elapidae and Hydrophidae venoms) and II (Crotalidae and Viperidae venoms) [20]. A few PLA2s of group IIA are noncovalent dimers formed by the enzymatically active subunit and inactive subunit, HDP-I and HDP-II from *Vipera nikolskii* venom being the examples of such dimers [21]. Previous studies have reported that PLA2s have a variety of biological activities, including anticancer [22,23], antibacterial [24], and antiviral ones [25,26,27]. 

Earlier, it was shown that PLA2s isolated from bee and snake venoms block HIV replication, preventing the intracellular release of the viral nucleocapsid protein [28], the effect being independent of the catalytic PLA2 activity. However, for human PLA2s, antiviral activity against Lentiviruses has been shown [29], which depended on the catalytic function and was mediated through the destruction of the viral membrane.

In this study, to clarify the role of phospholipolytic activity in antiviral effects, we analyzed the action of several snake PLA2s against HIV. It was found that the mechanisms of antiviral activity of the studied PLA2s were similar to the action of mammalian PLA2 but differed from the previously discovered mechanisms of bee and snake PLA2s. Our results indicate that snake PLA2s are capable of inhibiting syncytium formation between chronically HIV-infected cells and healthy CD4-positive cells and block HIV binding to cells. However, only dimeric PLA2s had pronounced virucidal and anti-HIV activity, which depended on their catalytic activity. The ability of snake PLA2s to inactivate the virus represents a novel defense mechanism that may provide an additional barrier to HIV infection.

## 2. Results

### 2.1. PLA2s from Snake Venoms

Several snake venom PLA2s and their subunits were used in this study. Two PLA2s of group IA were isolated from the venom of the krait *Bungarus fasciatus*: BF-PLA2-1 (basic phospholipase A2 1; UniProtKB Q90WA7) and BF-PLA2-II (phospholipase A2 II; GenBank AAK62361.1). Other utilized PLA2s were from group IIA: Vur-PL2 (UniProtKB F8QN53) from the viper *Vipera ursinii renardi* and HDP-1 and HDP-2 from the viper *V. nikolskii*. The last two dimers were composed of the HDP-1P (UniProtKB Q1RP79) and HDP-2P (UniProtKB Q1RP78) subunits, respectively, possessing phospholipolytic activity, combined with the inactive HDP-1I (UniProtKB A4VBF0) common for both dimers. HDP-2P and HDP-1I were obtained from HDP-2 using reverse-phase chromatography. The phospholipolytic activity of BF-PLA2-II was blocked by treatment with 4-bromophenacyl bromide, which selectively alkylates the His residue in the active site. A similar treatment of HDP-2P resulted in an inactive version of HDP-2P (called HDP-2Pinact). The mass spectrometry analysis revealed the incorporation of one 4-bromophenacyl residue into the HDP-2Pinact molecule. The phospholipolytic activity of HDP-2Pinact decreased by about 2200-fold (Figure S1).

### 2.2. Antiretroviral Activity of Snake Venom PLA2s

**Figure 1:**
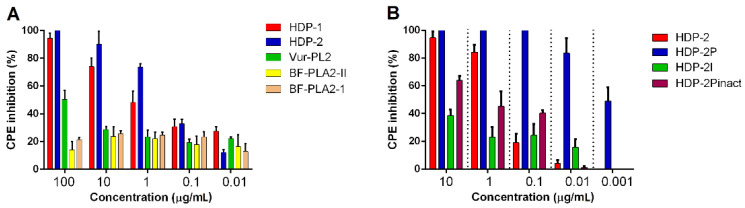
The antiviral activity of PLA2s against HIV-1 IIIB. PLA2s at different concentrations were added to MT-4 cells, followed by HIV-1 inoculation at 100 TCID_50_ (fifty percent tissue culture infective dose). Inhibition of the cytopathic effect (CPE) was determined using the methylthiazolyldiphenyl-tetrazolium bromide (MTT) test 5 days after infection. (**A**) Activity of five investigated PLA2s against HIV-induced CPE. (**B**) Antiviral activity of dimeric HDP-2 and its subunits against HIV-1. Results are representative of at least three independent experiments with the mean ± SEM.

The antiviral effect of various PLA2s was assessed against the reference strain HIV-1 IIIB using MT-4 cells. Monomeric groups IA BF-PLA2-II and BF-PLA2-1 showed no activity against HIV-1 (Figure 1A). Monomeric Vur-PL2 (group IIA) showed moderate activity against HIV-1 IIIB, inhibiting infection by ~50% at 100 μg/mL. Dimeric PLA2s HDP-1 and HDP-2 were found to inhibit HIV-1 IIIB replication with the IC_50_ values of 0.67 (24.8 nM) and 0.28 µg/mL (9.2 nM), respectively (Figure 1A,B and Table 1). All investigated PLA2s showed no cytotoxicity toward MT-4 cells at a concentration up to 100 μg/mL (37 µM, data not shown).

To examine whether the catalytic activity of PLA2s is required for inhibitory action, an enzymatically active subunit HDP-2P (from dimeric HDP-2) and enzymatically inactive subunits HDP-1I and HDP-2Pinact were studied. The enzymatically active HDP-2P markedly inhibited HIV-1 replication, and its activity was 30 times higher than that of dimeric HDP-2 (Figure 1B and Table 1). HDP-1I showed very weak activity against HIV-1 (Figure 1B). Inhibition of the enzymatic activity of HDP-2P (resulting in HDP-2Pinact) led to a considerable decrease in the antiviral effect (more than two orders of magnitude compared to HDP-2P), but this inactivated subunit was about three times more active than the dimeric HDP-2 (Figure 1B and Table 1).

**Figure 2:**
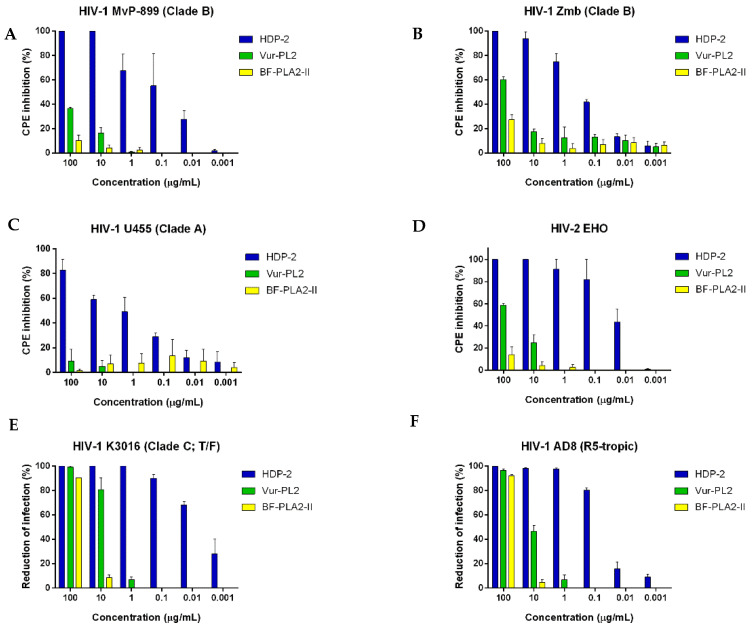
Antiviral activity of PLA2s against different strains and molecular clones of HIV. (**A**–**D**) PLA2s at different dilutions were added to the MT-4 cells; after which, the cells were infected with the corresponding HIV strain at 100 TCID_50_. CPE inhibition was determined after 48–72 h of infection using the MTT method. (**E**,**F**) The inhibition of the replication of infectious molecular clones using PLA2s was determined on TZM-bl cells after 48 h of infection by the activity of the β-Gal reporter gene. Data are the mean ± SEM (*n* = 3).

The antiviral activity of dimeric PLA2 HDP-2, as well as of the two monomeric Vur-PL2 and BF-PLA2-II, was studied using a panel of laboratory highly pathogenic HIV-1 strains of various subtypes: the HIV-2 EHO variant and infectious molecular clones (K3016 and AD8). It was found that HDP-2 strongly inhibited all HIV variants (Figure 2 and Table 2). The IC_50_ values for this PLA2 were in the range of 0.9–0.09 μg/mL. Monomeric PLA2 Vur-PL2 showed low activity against the HIV-1 MvP-899, HIV-1 Zmb, and HIV-2 EHO strains at the maximum concentration used in the experiments (100 μg/ml) (Figure 2A,B,D). However, both Vur-PL2 and BF-PLA2-II were able to inhibit almost completely the replication of infectious molecular clones K3016 and AD8 (Figure 2E,F and Table 2).

**Figure 3:**
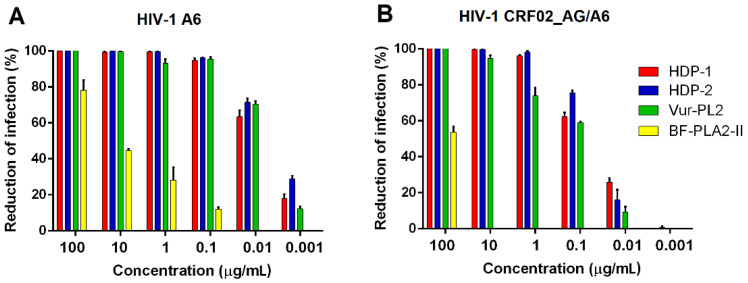
Antiviral activity of various PLA2s against the HIV-1 pseudoviruses. Various concentrations of PLA2s were added to TZM-bl target cells and transduced with pseudotyped HIV-1 virions, representing (**A**) sub-subtype A6 or (**B**) the recombinant form of CRF02_AG/A6. Infectivity was assessed with a β-Gal reporter gene activity after 48 h of infection. Data are the mean ± SEM from triplicates.

The antiviral effects of PLA2s were next tested on TZM-bl target cells by measuring the enhancement of β-Gal reporter gene activity by the HIV-1 pseudoviruses, representing sub-subtype A6 and recombinant form CRF02_AG/A6. All the examined PLA2s showed high activity against the HIV-1 pseudovirus representing the A6 sub-subtype (Figure 3A). The antiviral effect of PLA2s was significantly reduced when tested against the HIV-1 pseudovirus, representing a recombinant form of CRF02_AG/A6 (Figure 3B and Table 3). 

### 2.3. Effect of PLA2s on Syncytium Formation in Co-Cultures of HIV-1 Chronically Infected and Sup-T1 Cells

**Figure 4:**
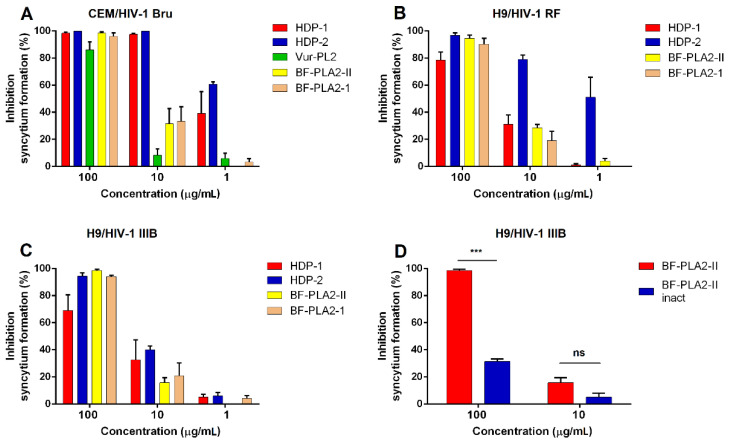
Effect of PLA2s on syncytium formation. Sup-T1 cells and HIV-1 chronically infected cells were mixed and treated with PLA2s. The levels of cell fusion were estimated after 24 h under a microscope. The inhibition of syncytium formation by PLA2s is shown for (**A**) CEM cells infected with HIV-1 Bru, (**B**) H9 cells infected with HIV-1 RF, and (**C**) H9 cells infected with HIV-1 IIIB. (**D**) The blockage of BF-PLA2-II enzymatic activity resulted in the decrease of the inhibition of syncytium formation by H9 cells infected with HIV-1 IIIB. The data represent the mean ± SEM from three independent experiments. Student’s *t*-test: *** *p* < 0.001, ^ns^ *p* > 0.05.

HIV-l-infected H9/HIV-1 IIIB, H9/HIV-1 RF, and CEM/HIV-1 Bru cells were used as inducer cells for syncytium formation. Uninfected CD4^+^ Sup-Tl cells were used as target cells. Of all the studied PLA2s, only dimeric HDP-1 and HDP-2 showed high activity, dose-dependently inhibiting the syncytium formation of cells chronically infected with various strains of HIV-1 (Figure 4). Monomeric Vur-PL2 has shown activity in blocking cell fusion induced by CEM/HIV-1 Bru cells (Figure 4A), but no activity was observed for other cells chronically infected with HIV-1. The PLA2s BF-PLA2-II and BF-PLA2-1 showed high activity at 100 μg/mL for all cell systems (Figure 4A–C). However, after the treatment of BF-PLA2-II with p-bromophenacyl bromide, which is an efficient inhibitor of PLA2s, a strong decrease in the inhibition of syncytium formation using H9/HIV-1 IIIB cells was observed (Figure 4D). Among the three HIV strains tested, HIV-1 IIIB preserved the highest capacity to induce syncytium formation in the presence of PLA2s. 

### 2.4. Dimeric PLA2s Possess Virucidal Activity and Block HIV-1 Adsorption

**Figure 5:**
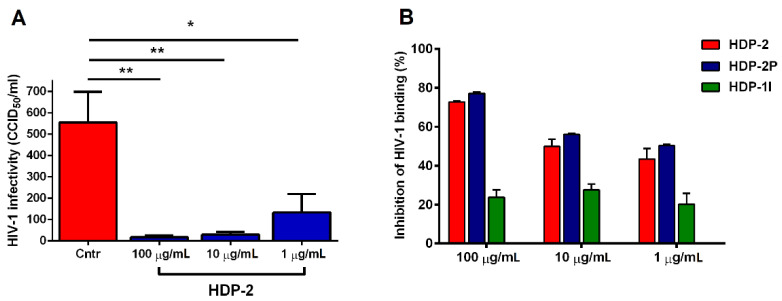
Virucidal activity of HDP-2 and the blocking of HIV-1 adsorption. (**A**) The HIV-1 stock was treated with different concentrations of HDP-2 and then used to infect MT-4 cells for 5 days. The antiviral effect of HDP-2 was assessed by determining the titer of the virus based on the CPE. Data represent the means ± SEM for three independent experiments. One-way ANOVA with Tukey’s post hoc test: * *p* ˂ 0.05 and ** p ˂ 0.01. (**B**) Inhibitory effects of HDP-2 and its subunits on HIV-1 IIIB binding to MT-4 cells. Data are presented as the mean ± SEM (*n* = 3).

We tested the possible direct virucidal activity of dimeric HDP-2 against HIV-1. For this, HIV-1 IIIB was incubated with various concentrations of HDP-2 or the control medium at 37 °C for 1 h and then diluted below the IC_50_ and inoculated into MT-4 cells by the limited dilution method. The results showed that HDP-2 effectively neutralized the infectivity of HIV-1 in a dose-dependent manner (Figure 5A). A significant inhibition of HIV-1 replication was observed when the virus was treated with HDP-2 concentrations of 100 (37 µM) and 10 μg/mL (3.7 µM).

Additionally, the activity of HDP-2 and its subunits against HIV-1 was analyzed in the virus adsorption assay. MT-4 cells were infected with HIV-1 IIIB for two hours in the presence of various dilutions of PLA2s or PBS as the control; after which, the intracellular concentration of the HIV-1 p24 antigen was determined by ELISA. The enzymatically inactive subunit HDP-1I showed weak activity in inhibiting the adsorption of HIV-1. However, HDP-2, as well as its catalytic subunit HDP-2P, blocked the binding of the virus to cells equally effective (Figure 5B).

### 2.5. Synergistic Activity of HDP-2 and HIV Nucleoside Reverse Transcriptase Inhibitors (NRTIs)

**Figure 6:**
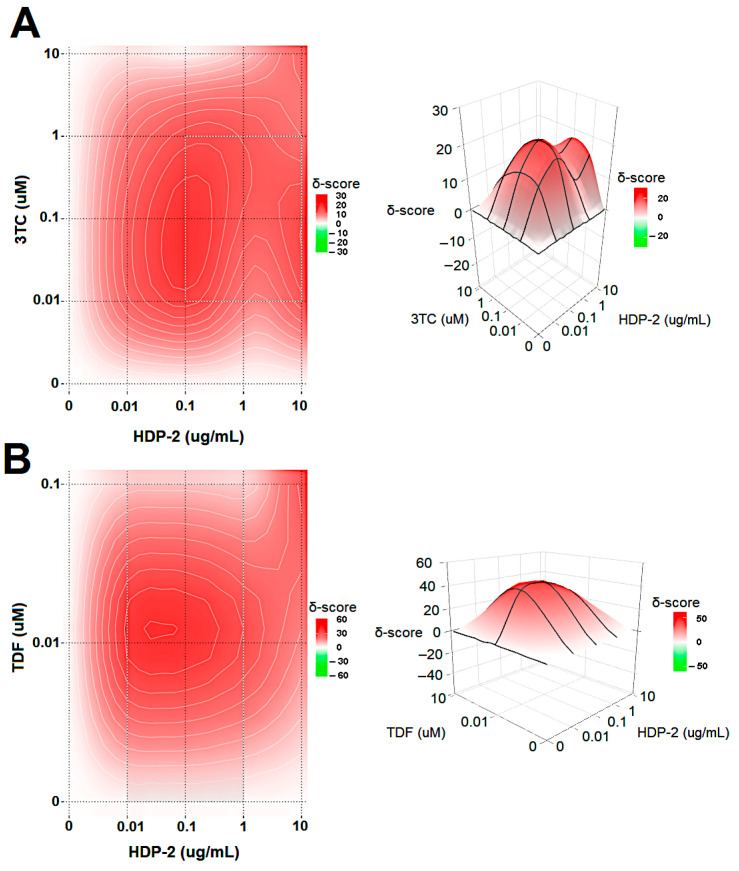
Combinations of HDP-2 with (**A**) 3TC or (**B**) TDF inhibit HIV-1-mediated CPE in MT-4 cells. The interaction landscapes of the drug combinations are presented as dose–response matrices. Synergy distribution and 3D synergy landscapes are shown. The most synergistic area is displayed on the matrices.

We tested combinations of HDP-2 with Lamivudine (3TC) or Tenofovir disoproxil fumarate (TDF) against HIV-1-mediated CPE in MT-4 cells. Different concentrations of the compounds alone or in combinations were added to uninfected or virus-infected cells. HIV-induced CPE were measured after 48 h to determine the compound synergy. Our data identified that a combination HDP-2/3TC and HDP-2/TDF suppressed HIV-1-mediated CPE with synergy scores of 12.9 and 24.5, respectively (Figure 6). Thus, the two investigated drug combinations showed a pronounced synergy, with a higher synergy score for the HDP-2/TDF combination. 

## 3. Discussion

In modern classification, there are two main types of HIV, namely HIV-1 and HIV-2. These viruses presumably arose as a result of the independent transmission of SIV (Simian Immunodeficiency Virus) to humans by chimpanzees and mangabeys, respectively [1]. HIV-2 is known to be less pathogenic and less likely to be transmitted than HIV-1. The HIV-1 species is classified into a major group M and several subsidiary groups. It is believed that the M, N, O, and P groups were formed as a result of independent cases of SIV transmission from monkeys to humans and the subsequent mutation of the virus to HIV. Group M viruses (from the English “Main”) are the cause of more than 90% of HIV infections. Group M is classified into several clades, called subtypes, also denoted by letters. For example, subtype A is widespread in West Africa and Russia. The HIV subtypes change dynamically, posing challenges for diagnosis and treatment, as well as for vaccine design and development [30].

It was shown earlier that PLA2s possessed inhibitory activity against the HIV virus. The first study appeared more than 20 years ago and showed that four secretory PLA2s from animal venoms were very potent HIV-1 inhibitors [28]. No influence of PLA2s on virus binding to cells or on syncytia formation were found, and PLA2 catalytic activity was not shown to be involved in the antiviral effect. The authors assumed that the antiviral activity involved a specific interaction of PLA2s with host cells. Later, this finding was supported by the data for the CM-II isoform of secreted PLA2 obtained from *Naja mossambica mossambica* snake venom [25].

However, in contrast to these results, it was found that a secretory human sPLA2-X neutralized HIV-1 through degradation of the viral membrane. The PLA2 antiviral activity depended on catalytic function, and the target cells of infection were unaffected [29]. Besides, in patient plasma, PLA2 of group IB (PLA2G1B) was identified; PLA2G1B synergized with the HIV gp41 envelope protein, and the PLA2G1B/gp41 pair induced CD4+ T-cell unresponsiveness (anergy) [31].

In our work, we tried to find out how the snake PLA2 acted on HIV or its target cells. As discussed above, HIV-1 is incredibly adaptive and diverse, and its recombinant circulating forms have appeared recently, which were studied in this work as well. Besides, we have extended the array of PLA2s studied. The snake venom PLA2s investigated earlier included mainly those from the D49 sub-subfamily of Group I: NmmCMIII from *N. mossambica mossambica*, OS1 and taipoxin from *Oxyuranus scutellatus scutellatus*, nigexine from *N. nigricollis* [28], and CM-II isoform from *N. m. mossambica* [25]. In addition, bee venom PLA2 of Group III and BaIV PLA2-like protein from *Bothrops asper* of the K49 sub-subfamily from Group II were studied [28]. In our work, we used dimeric phospholipases HDP-1 and HDP-2 and their subunits HDP-1I and HDP-2P from *V. nikolskii*, as well as Vur-PL2 from *V. ursinii renardi*; all these PLA2s are from the D49 sub-subfamily from Group II. Moreover, we studied BF-PLA2-1 and BF-PLA2-II from krait *B. fasciatus*. Although these PLA2s belong to the D49 sub-subfamily of Group I, they are from a snake of the *Bungarus* genus, which is quite different from the *Naja* genus studied earlier. 

We evaluated the ability of snake venom PLA2s to inhibit HIV-1 replication and found that dimeric PLA2s exhibit high antiviral activity against both wild-type HIV-1 and of pseudoviruses of different subtypes. The studied PLA2s are capable of inhibiting distinct HIV-1 subtypes in different ways. A high antiviral effect was observed against the A6 sub-subtype, while a significant decrease in the antiviral activity was observed for the recombinant CRF02_AG/A6 form. Such a distinction may be explained by the differences in the structures of the virion glycoproteins, a different degree of glycosylation, or alterations in the composition of the viral membrane. Previously, it was reported [32] that HIV-1 could escape the inhibitory effect of PLA2s by selecting a specific envelope glycoprotein (presence of rare mutations in the N-terminal region and V1–V2 envelope loop extensions), which allowed the virus to infect cells via an alternative pathway of entry based on the transfer of endosomes. Thus, the observed differences in the antiviral effect of the PLA2s, detected on pseudoviruses of two different subtypes, may also arise from the presence of mutations in HIV-1 glycoproteins. However, dimeric PLA2 HDP-2 has shown a broad spectrum of anti-HIV activity against viruses of different subtypes and possessing different coreceptor tropisms.

In the previous studies, PLA2 isolated from the venom of *Crotalus durissus terrificus* has been shown to have potent antiviral activity against the Dengue virus (DENV-2) and Yellow fever virus (YFV) in a virucidal assay, providing compelling evidence for the direct action on the viral particle [26,33]. In our recent work, we demonstrated that dimeric HDP-2 isolated from the viper *V. nikolskii* possessed potent virucidal effects, which were related to its phospholipolytic activity, and inhibited cell–cell fusion mediated by the SARS-CoV-2 spike glycoprotein [34]. Here, we also found a direct virucidal effect of HDP-2, which confirmed the ability of PLA2s to inactivate various viruses. The inhibition of viruses is most likely caused by the enzymatic activity of PLA2, which is based on the hydrolysis of the sn-2-acyl ester linkage of sn-3 glycerophospholipids, producing fatty acids and lysophospholipids [35]. The hydrolysis of glycerol phospholipids can lead to degradation of the viral envelope, which will lead to the loss of infectivity of virions and of their ability to infect cells.

Several sPLA2s, including those from bee and snake venoms, have been reported to protect primary human blood lymphocytes from the replication of various macrophage and T-cell tropic HIV-1 strains [28]. However, it was found that sPLA2s inhibited HIV-1 not through a virucidal action. No influence either on the binding of the virus to cells or on the formation of syncytia was found, and the catalytic activity of sPLA2 was not involved in the antiviral effect. In contrast, we found that the enzymatic activity of the snake PLA2s analyzed in our work is essential for the antiviral effect. Inhibition of the enzymatic activity of the catalytic subunit HDP-2P, derived from dimeric HDP-2, led to a sharp decrease in anti-HIV activity. This finding is consistent with a previous report, showing that human sPLA2-X has antiviral activity against lentiviruses due to its catalytic function and viral envelope recognition [29]. The discrepancies in the results reported earlier [28] and ours could arise from the differences in methodology and experimental design. In the cited work [28], single-round infection assays were used to determine the PLA2 antiviral properties, while we used infectious viruses for multicycle infection. To assess the virucidal activity, only bee venom PLA2 belonging to group III was used in Reference [28], while PLA2s from groups I and II were utilized in our work. The same is true for binding of the virus to the cell and the formation of syncytia. As for the role of the catalytic activity, again, the effect of treating only bee venom PLA2 with inhibitors was studied in work [28]. Moreover, no data about the phospholipolytic activity of inhibited PLA2 was given. Since group III PLA2s from one site and groups I and II PLA2s from other the site differ significantly in their structures and substrate specificities, it is quite possible that the molecular mechanism underlying the anti-HIV effects are different for these groups. 

Our results show that the PLA2 inhibition of HIV-1 occurs early in the viral replication cycle. HIV enters the cell by fusion at the plasma membrane, a process triggered by the binding of gp120 to the CD4 and chemokine receptors. The data obtained show that the binding of gp120 to these cellular receptors is inhibited by PLA2s, since all studied PLA2s exhibited activity in the inhibition of syncytium formation in the system of the cocultivation of HIV-1 chronically infected cells with CD4^+^ Sup-T1 cells. In addition, we found that dimeric HDP-2, as well as its enzymatically active subunit HDP-2P, are capable of inhibiting HIV-1 binding to cells. Similar data have been shown earlier for the crude *C. d. terrificus* venoms and isolated toxins such as crotoxin, PLA2-CB, and PLA2-IC, which inhibited the adsorption of both YFV and DENV-2 [33]. Interestingly, PLA2s isolated from the bee venom showed no inhibitory effect on the syncytium formation, suggesting that bee PLA2s do not act on the virus–cell fusion process [28]. However, synthetic peptides derived from bee venom PLA2 were able to inhibit HIV-1 replication and syncytium formation by interacting with the cellular coreceptor CXCR4, which may be due to the presence of homologous sequences in PLA2 and viral envelope glycoproteins [36]. 

## 4. Materials and Methods

### 4.1. Cells and Viruses

Human T-cells H9, MT-4, Sup-T1 (NIH AIDS Research and Reference Reagent Program, ARRRP, Manassas, VA, USA), and CCRF-CEM (CCL-119, ATCC, Manassas, VA, USA) were cultivated in complete RPMI 1640 medium supplemented with 10% fetal bovine serum (FBS), 1× pen/strep, and 1× GlutaMAX (all from Gibco RBL Life Technologies, Waltham, MA, USA). HIV-1 IIIB, HIV-1 RF, and HIV-1 Bru were obtained from the ARRRP. Chronically infected H9/HIV and CEM/HIV cells were prepared by exposing H9 or CCRF-CEM cell cultures to HIV-1 IIIB, HIV-1 RF, or HIV-1 Bru for 4 weeks. Persistent virus production was assessed by HIV-1 p24 antigen ELISA (Vektor-Best, Novosibirsk, Russia). 293T (ATCC, CRL-3216) and TZM-bl cells (stably expressing CD4 and CXCR4, as well as CCR5, and containing HIV-1 tat-regulated reporter genes for luciferase and β-galactosidase (ARRRP) were cultivated in complete DMEM medium (Gibco RBL Life Technologies, Waltham, MA, USA). Virus stocks were prepared from the supernatant of acute infected MT-4 or CCRF-CEM cells. 

In some experiments, the following viral strains were used: HIV-1 Zmb (Clade B, Belarus) [37], HIV-1 U455 (Clade A, Uganda) [38], and HIV-1 MvP-899 (Clade B, Germany) [38]. The HIV-1 molecular clone K3016 (clade C)-transmitted founder virus and AD8 (R5-tropic) were obtained through ARRRP and generated by transfecting 293T cells [39].

### 4.2. Phospholipases A2

Phospholipase A2 Vur-PL2 (UniProtKB F8QN53) possessing an enzymatic activity of 3.6 mmol/min/mg was obtained from viper *V. ursinii renardi* venom as described [40]. Phospholipase A2 1 (BF-PLA2-1, UniProtKB Q90WA7) and phospholipase A2 II (BF-PLA2-II, GenBank AAK62361.1) were isolated from krait *Bungarus fasciatus* venom as described [23]. Dimeric phospholipases HDP-1 and HDP-2 were purified from viper *V. nikolskii* venom and separated into subunits HDP-1I (UniProtKB A4VBF0), HDP-1P (UniProtKB Q1RP79), and HDP-2P (UniProtKB Q1RP78) as described [21]. It was shown that HDP-1I was enzymatically inactive; HDP-1P and HDP-2P possess higher enzymatic activity (1.25 and 0.81 mmol/min/mmol of protein, respectively) than HDP-1 (0.56 mmol/min/mmol) and HDP-2 (0.31 mmol/min/mmol) [21]. To obtain inactivated HDP-2P, 4-bromophenacyl bromide (Lancaster Synthesis, Morecambe, England) solution in acetone was added (final concentration of 200 µM) to a 20-µM HDP-2P solution in 50-mM Tris HCl buffer (pH 7.5) containing 10-mM Na_2_SO_4_. After incubation for 6 h at room temperature, the mixture was separated on a Jupiter C18 HPLC column (Phenomenex, Torrance, CA, USA) using an acetonitrile gradient from 20 to 50% in 30 min in the presence of 0.1% trifluoroacetic acid. The phospholipolytic activity was determined using synthetic fluorescent substrate 1-palmitoyl-2-(10-pyrenyldecanoyl)-sn-glycero-3-phosphocholine (Molecular Probes, Waltham, MA, USA) (according to Radvanyi et al. [41]) and a Hitachi F-4000 spectrofluorometer (Hitachi, Tokyo, Japan).

Inactivation of the enzymatic activity of BF-PLA2-II was performed as described previously [42]. Briefly, 3 mg of BF-PLA2-II were dissolved in 1 mL of 0.1-M Tris and 0.7-mM EDTA (pH 8.0) buffer. Then, 4-bromophenacyl bromide (1.5 mg/mL in ethanol, with a final concentration of 0.2 mg/mL) were added and incubated at room temperature for 24 h.

### 4.3. Anti-Retrovirus Assay

The anti-HIV assays were done as described previously [43]. Briefly, MT-4 cells (2 × 10^4^/well) were seeded in 96-well plates. Then, cell suspension was mixed with the appropriate solution of PLA2 (i.e., final concentrations of 100, 10, 1, 0.1, 0.01, and 0.001 µg/mL) and infected with HIV-1 IIIB at 100-fold the 50% cell culture infective dose (CCID_50_). After 5 days, the viability of mock, PLA2-treated, and HIV-infected cells was examined spectrophotometrically by the methylthiazolyldiphenyl-tetrazolium bromide (MTT) assay. The 50% inhibitory concentration (IC_50_) required to prevent an HIV-induced cytopathic effect (CPE) was determined by a regression analysis using GraphPad Prism 6 (GraphPad Software Inc., La Jolla, CA, USA).

### 4.4. Syncytium Formation Assay

The syncytium assay was performed by cocultivating equal numbers (8 × 10^4^) of chronically infected H9/HIV-1 IIIB, H9/HIV-1 RF, or CEM/HIV-1 Bru cells with CD4^+^ Sup-T1 cells in 96-well plates in the presence of different concentrations of PLA2. After 24 h of cocultivation, syncytium formation was scored visually under a microscope.

### 4.5. Virucidal Assay

Stock of infectious HIV-1 IIIB was mixed with various concentrations of PLA2 and incubated for 1 h at 37 °C. Samples were diluted 5000 times with complete RPMI 1640 medium to reach a concentration of PLA2 below the IC_50_. Then, the infectivity of HIV-1 IIIB was determined by titration on MT-4 cells. After 5 days, the cells were observed under microscope, and the CCID_50_ value was calculated using the Reed and Muench method [44].

### 4.6. Virus Adsorption Assay

In this assay, the inhibitory effects of PLA2 (HDP-2, HDP-2P, and HDP-2I) on HIV-1 IIIB virus adsorption into MT-4 cells were measured as previously described [45]. MT-4 cells (10^6^ cells/mL) were incubated with HIV-1 at 1000 CCID_50_ in the absence or presence of serial dilutions of PLA2. After 2 h of incubation at 37 °C, the cells were washed with PBS to remove the unabsorbed virus. Then, the cells were lysed with PBS containing 0.01% Triton X-100. The amount of p24 antigen was determined by the HIV-1 p24 antigen ELISA (Vektor-Best, Novosibirsk, Russia).

### 4.7. Construction and Production of HIV-1 Env-Functional Clones

The construction of HIV-1 pseudovirions was performed as described previously [46]. Briefly, RNA isolated from the serum samples of HIV-infected patients was initially amplified by a reverse transcription polymerase chain reaction (RT-PCR) and sequenced in fragments corresponding to the HIV-1 genome-encoding protease and reverse transcriptase [47]. Viruses of sub-subtype A6 and CRF02_AG/A6 were selected for envelope (*env*) gene amplification and cloning. Amplified fragments of the full-length *env* gene were extracted from agarose gel with the MinElute Gel Extraction kit (Qiagen, Valencia, CA, USA) according to the manufacturer’s instructions. To prepare *rev*/*env* expression plasmid, the obtained DNA was ligated in a pcDNA3.1/V5-His-TOPO TA expression vector (Invitrogen, Carlsbad, CA, USA). 

*Env*-pseudoviruses were prepared by transfecting 293T cells (5 × 10^6^ cells in 10 mL growth medium in a 100-mm Petri dish) with 4 μg of *rev*/*env* expression plasmid and 8 μg of an *env*-deficient HIV-1 backbone vector (pSG3Δenv) using Lipofectamine 2000 (Invitrogen, USA), as described by the manufacturer. After 48 h, pseudovirus-containing culture supernatants were harvested, filtered (0.45 μM), and stored at −80 °C in 1-mL aliquots. *Env*-pseudotyped virus stocks were titrated as described previously [48]. Briefly, TZM-bl cells were seeded in a 96-well plate and infected with a 10-fold serial dilution of the virus stock. After 48 h of viral infection, cells were fixed in 0.1% paraformaldehyde for 5 min at room temperature. The cells were then washed three times with PBS and stained with staining solution containing 400 μg/mL of X-gal (5-bromo-4-chloro-3-indolyl-β-D-galactopyranoside), 4-mM MgCl_2_, 4-mM potassium ferrocyanide, and 4-mM potassium ferricyanide in PBS for 2 h at 37 °C. The stained blue colonies were then visualized and counted under a microscope.

### 4.8. Drug Susceptibility Assay against of HIV-1 Env-Functional Clones

The drug susceptibility of HIV-1 was determined by an enzymatic assay of β-galactosidase activity using TZM-bl cells expressing CD4 and coreceptors CXCR4 and CCR5. For this, the TZM-bl cells were seeded in 96-well plates (10^4^/well) the day before the experiment. Then, various concentrations of PLA2s were added to the cells, followed by inoculation with HIV-1 pseudoviruses at 500 blue cell-forming units (BFU)/well. After 48 h of inoculation with HIV-1, the cells were lysed with PBS containing 1% Triton-X100 and incubated at 37 °C for 1 h with 10-mM chlorophenol red-β-D-galactopyranoside (CPRG; Sigma, St. Louis, MO, USA) in 2-mM MgCl_2_ and 100-mM KH_2_PO_4_. The reaction was stopped by adding 0.5-M Na_2_CO_3_. Optical density at 570 nM was measured on a microplate reader (Hidex Sense Beta Plus, Hidex, Turku, Finland). Drug concentrations that brought about 50% inhibition of the β-galactosidase activity were determined.

### 4.9. Analysis of Drug Combination and Synergy

The combinational inhibitory activity of PLA2 HDP-2 and Lamivudine (3TC) or Tenofovir disoproxil fumarate (TDF) against HIV-1 was analyzed using the Zero Interaction Potency (ZIP) reference model by SynergyFinder version 2 [49]. MT-4 cells were treated with different concentrations of PLA2 and the corresponding drug and infected with HIV-1 IIIB (100 TCID_50_) or the mock. After 72 h, virus-induced CPE was measured using the MTT method. The percent of HIV-1 inhibition resulting from the combination of HDP-2/3TC or HDP-2/TDF was assessed for synergistic action based on synergy scores from a dose–response matrix. 

## 5. Conclusions

Our data showed that dimeric PLA2s can inhibit HIV-1 replication with high potency. However, the new HIV-1 strains (forms), as follows from our present work, were less sensitive to the PLA2 effects. The destructive effect of PLA2s on the viral membrane was confirmed using a virucidal test. PLA2 can exert a direct effect on the viral particles, most likely due to the cleavage of glycerophospholipids on the viral envelope, which can lead to the destruction of the lipid bilayer and destabilization of the virus. In addition, PLA2 HDP-2 inhibits the early stages of the HIV-1 replication cycle, which was confirmed using inhibition assays of syncytium formation and viral adsorption. In addition, we showed for the first time the synergistic effects of PLA2 HDP-2 with HIV NRTIs such as Lamivudine and Tenofovir. Our data clearly indicated that PLA2s exerted their effects by direct action on the virus and not on the target cells.

## Figures and Tables

**Table 1:** IC_50_ values for dimeric PLA2 HDP-2 and its subunits.

MT-4/HIV-1 IIIB	IC_50_ (µg/mL) with 95% CI *
HDP-2	0.25 (0.18–0.35)
HDP-2P	0.0008 (0.0005–0.001)
HDP-2I	>10
HDP-2Pinact	0.1 (0.04–0.3)

* CI: confidence intervals.

**Table 2:** IC_50_ values for the three investigated PLA2s in different virus–cell systems.

Cell/Virus System	IC_50_ (µg/mL) with 95% CI *		
	HDP-2	Vur-PL2	BF-PLA2-II
MT-4/HIV-1 MvP-899	0.1 (0.006–0.01)	- **	-
MT-4/HIV-1 Zmb	0.22 (0.15–0.32)	16.45 (3.9–68.1)	-
MT-4/HIV-1 U455	0.9 (0.32–2.6)	-	-
MT-4/HIV-2 EHO	0.016 (0.008–0.03)	~10.41	-
TZM-bl/HIV-1 K3016	0.009 (0.006–0.015)	4.37 (3.2–5.9)	~12.21
TZM-bl/HIV-1 AD8	0.046 (0.037–0.057)	10.29 (8.8–11.9)	~14.26

* CI: confidence intervals; **: not detected.

**Table 3:** IC_50_ values for the four PLA2s investigated in pseudovirus systems.

PLA2	IC_50_ (µg/mL) with 95% CI *	
	HIV-1 A6	HIV-1 CRF02_AG/A6
HDP-1	0.009 (0.008–0.01)	0.046 (0.038–0.056)
HDP-2	0.008 (0.007–0.009)	0.04 (0.034–0.047)
Vur-PL2	0.007 (0.006–0.01)	0.1 (0.07–0.16)
BF-PLA2-II	3.22 (1.78–5.84)	~31.75

* CI: confidence intervals.

## Data Availability

All data obtained in this study are contained within the article.

## Supplementary Materials

The following supporting information can be downloaded at https://www.mdpi.com/article/10.3390/ijms23031610/s1.
