# Supplementary material for: Anti-HIV Activity of Snake Venom Phospholipase A2s: Updates for New Enzymes and Different Virus Strains
Source: Int J Mol Sci. 2022 Jan 30;23(3):1610. doi: 10.3390/ijms23031610 (PMC8835987; doi:10.3390/ijms23031610)
Supplement: Supplementary file 1 [file ijms-23-01610-s001.zip › ijms-1554032-supplementary.pdf]

## Supplemental Information

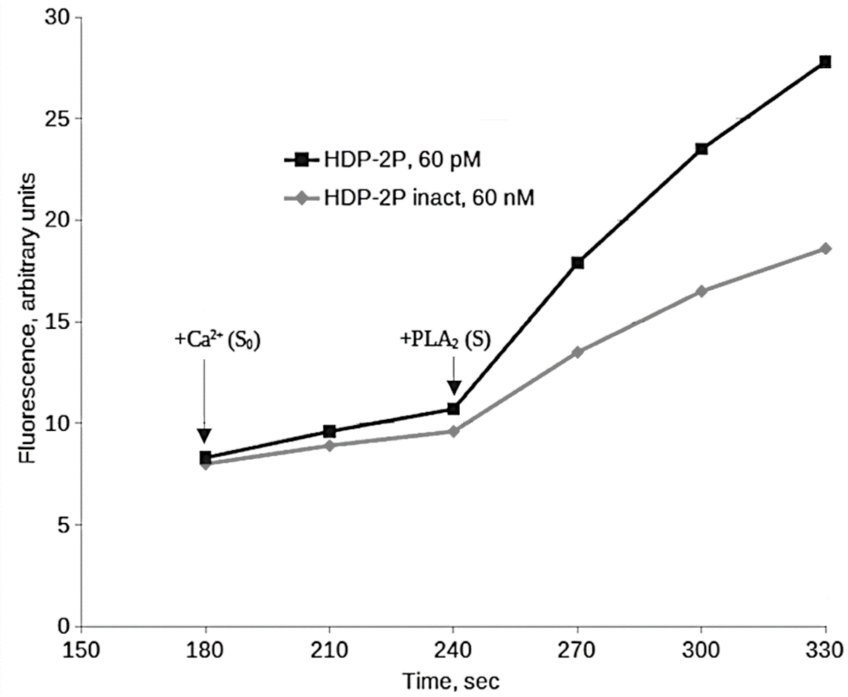


**Figure S1. Determination of phospholipolytic activity of HDP-2P (black squares) and HDP-2P inact (gray rhombuses).** The determination was done as previously described [Radvanyi at al.,1989]. Fluorescence was measured every 30 s. During the first 180 s the level of spontaneous hydrolysis of the fluorescent substrate prior to the addition of calcium chloride was established. The data were obtained at concentrations of 60 pM and 60 nM for HDP-2P and HDP-2P inact, respectively. The activity (micromoles per minute) was calculated by the formula:

$$A=2\times10^{-4}\times(S-S_0)\times V/F_{max},$$

where S is the slope of the curve representing the increase of fluorescence versus time (in s); $S_0$ is the slope observed in the absence of enzyme; V is the volume (in µl) of pyrene phospholipids (0.2 mM) in the reaction medium; and $F_{max}$ is the maximal fluorescent signal with complete hydrolysis of the substrate. Both V and $F_{max}$ are equal for HDP-2P and HDP-2P inact, therefore for comparison of activities, we can compare $S-S_0$ values, which are 7.5 and 3.4 for HDP-2P and HDP-2P inact, respectively, i.e. the activity of 60 pM HDP-2P is higher than that of 60 nM HDP-2P inact by 2.2 fold. As the difference in concentration is the three orders of magnitude, HDP-2P inact is less active than HDP-2P by 2200 fold.
